# Impact of solvent choice during microfluidic manufacture on the in vivo performance of liposomal doxorubicin

**DOI:** 10.1007/s13346-026-02062-4

**Published:** 2026-03-02

**Authors:** Sarah Lindsay, René Holm, Sanket Shah, Yvonne Perrie

**Affiliations:** 1 Strathclyde Institute of Pharmacy and Biomedical Sciences, University of Strathclyde, 161 Cathedral St, Glasgow, G4 0RE UK; 2 Department of Physics, Chemistry, and Pharmacy, University of Southern Denmark, Campusvej 55, 5230 Odense, Denmark; 3 Therapeutics Development and Supply, Janssen Pharmaceutica NV, a Johnson & Johnson Company, Turnhoutseweg 30, 2340 Beerse, Belgium

**Keywords:** Liposomes, Liposomal doxorubicin, Microfluidic manufacture, Solvent effects, Biodistribution

## Abstract

Microfluidics offers a reproducible approach to liposome manufacture; however, the impact of solvent choice on formulation performance remains underexplored. Here, we investigated whether solvent selection during microfluidic manufacturing influences liposome performance by assessing its effect on the drug release and biodistribution of liposomal doxorubicin, using rotary evaporation as a conventional method of comparison. PEGylated liposomes (DSPC:Chol:DSPE-PEG2000, 3:1:1 w/w) were prepared using a staggered herringbone microfluidic mixer, with lipids dissolved in either ethanol or Transcutol, and compared with liposomes produced by rotary evaporation and extrusion. All formulations were actively loaded with doxorubicin via an ammonium sulphate gradient, purified by tangential flow filtration, and characterised for size, polydispersity, and drug loading. The three formulations (two microfluidic (ethanol vs Transcutol) and one rotary evaporation control) were intravenously administered to Sprague–Dawley rats (1 mg/kg), and doxorubicin concentrations in plasma and tissues were quantified using LC–MS. Although solvent choice produced liposomes with broadly comparable physicochemical properties (~ 100–120 nm, PDI < 0.25, encapsulation > 90%), Transcutol-based microfluidic liposomes showed prolonged and higher concentrations of doxorubicin within the plasma and tissue samples compared with liposomes produced by ethanol microfluidics and rotary evaporation. By contrast, the manufacturing method alone (microfluidics using ethanol as the solvent versus rotary evaporation) did not significantly influence biodistribution. These findings highlight solvent selection as an important parameter in microfluidic liposome manufacture, demonstrating that matching standard critical quality attributes and in vitro release behaviour alone may be insufficient to ensure comparable in vivo performance.

**Graphical abstract:**

**Figure:**
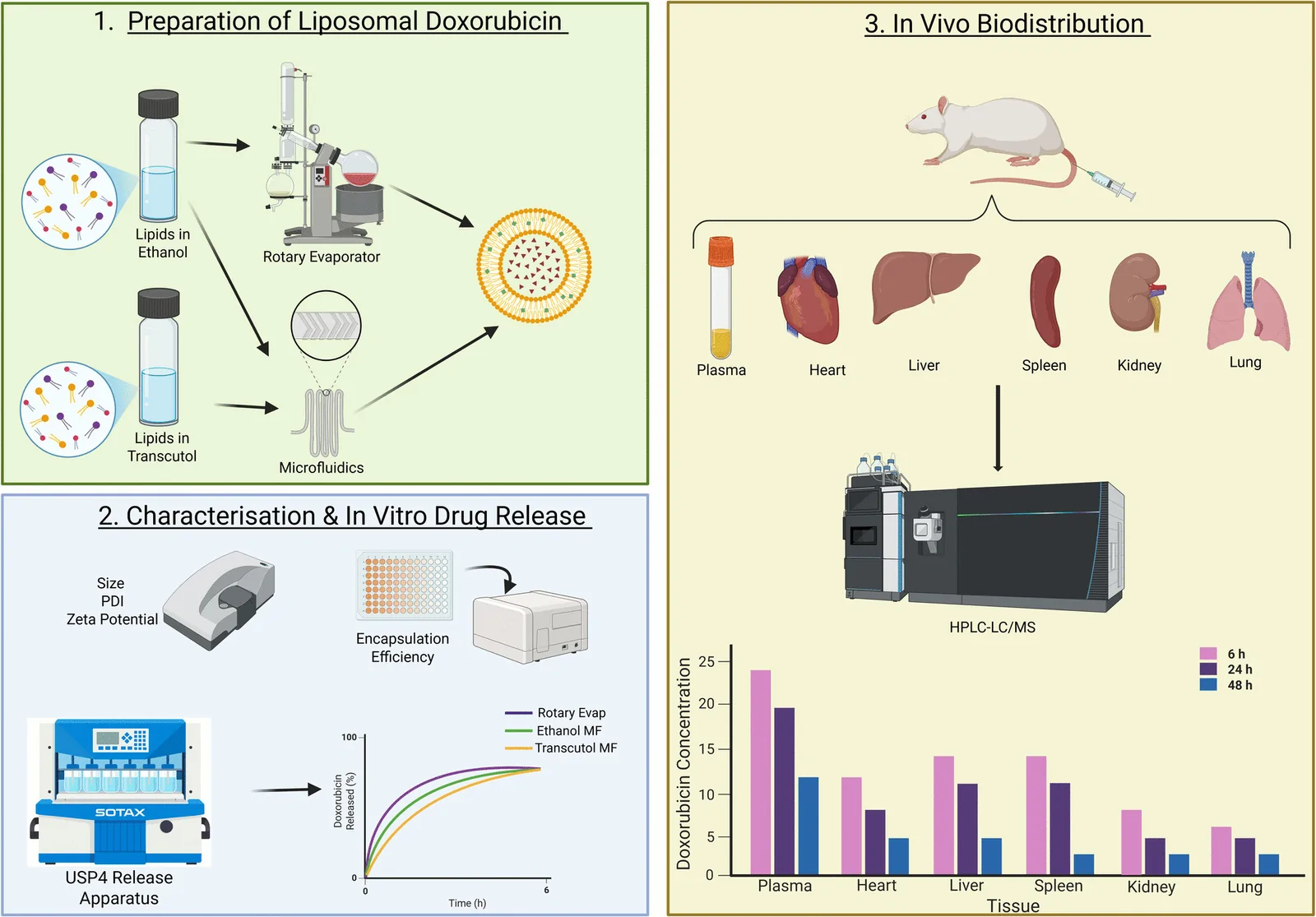

**Supplementary Information:**

The online version contains supplementary material available at https://doi.org/10.1007/s13346-026-02062-4.

## Introduction

Liposomes enhance drug delivery and bioavailability by encapsulating therapeutic agents within their aqueous core or lipid bilayer [1]. Their clinical relevance was established with the approval of the first FDA-approved nanomedicine, Doxil^®^/Caelyx (US/UK), a PEGylated “stealth” liposome introduced in 1995 [2]. The rational design of effective liposomal drug delivery systems relies on a clear understanding of how they interact with biological environments, from the site of administration to the target organ. These interactions govern biodistribution and influence circulation, clearance, and tissue uptake. Upon entry into the bloodstream, liposomes encounter plasma proteins and blood cells, interactions that can alter their pharmacokinetics and therapeutic performance [3].

In contrast to free drug molecules, and as exemplified by Doxil^®^, liposomal formulations reduce drug accumulation in healthy tissues and favour delivery to inflamed or tumour sites. Liposomal doxorubicin (Doxil^®^) was developed to increase therapeutic efficacy whilst also reducing toxicity to healthy tissues. PEGylation of the liposomes extends circulation time and reduces opsonisation by the mononuclear phagocyte system (MPS), enhancing passive targeting via the enhanced permeability and retention (EPR) effect [4]. Liposome size, charge and composition further influence clearance and biodistribution, with small PEGylated vesicles (< 100 nm) often being designed to evade rapid uptake and clearance [5, 6].

Despite being widely researched since their discovery in the 1960 s, very few liposomal drug formulations have reached clinical use, partly due to challenges associated with cost and scalable manufacturing. The production of liposomes generally relies on a ‘top-down’ multi-batch process in which large multilamellar vesicles are first generated and then subjected to size-reduction steps such as extrusion or sonication to produce more uniform unilamellar liposomes. While effective, these additional downstream processing steps increase cost and limit scalability [7–9]. Beyond these manufacturing challenges, there is growing recognition that equivalence in standard critical quality attributes and in vitro release, while necessary for quality control, may not on its own be sufficient to ensure comparable in vivo performance when alternative manufacturing routes or process conditions are employed, particularly for complex liposomal formulations where biological interactions can alter behaviour post-administration.

In response to these limitations, alternative manufacturing strategies have been explored. Bottom-up approaches assemble vesicles directly from molecular components, offering greater control over particle size and lamellarity and thereby eliminating the need for subsequent size-reduction steps [10]. Among these, microfluidic mixing is recognised as a particularly useful strategy, offering reproducibility and precise control over liposome characteristics. Microfluidic technologies are now widely applied across drug development, biomedical diagnostics, and the food and chemical industries [11, 12].

This study addresses two related objectives. First, it examines whether microfluidic manufacture can generate liposomes with performance comparable to those produced by conventional rotary evaporation, thereby supporting translation between manufacturing methods. Second, it evaluates whether solvent selection within microfluidic manufacture, specifically ethanol versus Transcutol, represents a critical material attribute influencing liposome performance. Liposomal doxorubicin is used as a benchmark, and performance is assessed using standard critical quality attributes, in vitro release, and in vivo biodistribution.

## Methods

### Materials

1,2-Dioctadecanoyl-sn-glycero-3-phosphocholine (DSPC; Avanti Polar Lipids, Alabaster, AL, USA), 1,2-distearoyl-sn-glycero-3-phosphoethanolamine-N-[methoxy(polyethylene glycol)−2000] (sodium salt) (DSPE-PEG2000; ≥ 99%; Lipoid, Ludwigshafen, Germany), and cholesterol (Chol; ≥ 99%; Sigma-Aldrich, Poole, UK) were used. Doxorubicin and daunorubicin were purchased from Fisher Scientific (Loughborough, UK), and ammonium sulphate ([NH_4_]_2_SO_4_; ≥ 99%) was obtained from Sigma-Aldrich (Poole, UK). Phosphate-Buffered Saline tablets (10 mM, PBS; pH 7.3) were purchased from Oxoid Ltd. (Basingstoke, UK). Ethanol and methanol (HPLC grade, ≥ 99.8%) were purchased from Sigma-Aldrich, and Transcutol (diethylene glycol monoethyl ether; general-purpose grade) was obtained from Fisher Scientific (Loughborough, UK).

### Preparation of ammonium sulphate–loaded PEGylated liposomes using microfluidics

PEGylated liposomes were prepared using a bench-scale microfluidic system (NanoAssemblr Benchtop, Precision NanoSystems Inc., Vancouver, Canada) equipped with a staggered herringbone micromixer (SHM). Lipid formulations contained DSPC, cholesterol (Chol), and DSPE-PEG2000 at a weight ratio of 3:1:1 (w/w) corresponding to an approximate molar ratio of 10.6:7.2:1 (DSPC:Chol:DSPE-PEG2000). A 20 mg/mL total lipid stock was prepared by solubilising the lipids in either ethanol or Transcutol; heating was required for solubilisation, but the solutions remained stable once dissolved. The aqueous phase consisted of 250 mM ammonium sulphate ([NH_4_]_2_SO_4_), unless otherwise stated. Liposomes prepared from the ethanol stock were manufactured at a total flow rate (TFR) of 10 mL/min with a flow rate ratio (FRR) of 1.5:1, while those prepared from the Transcutol stock were produced at a TFR of 5 mL/min and FRR of 1:1. Total flow rates for each solvent were selected based on a previously published design of experiments that identified solvent-specific conditions required to achieve comparable liposome sizes during microfluidic manufacture [13].

### Rotary evaporation production of PEGylated liposomes encapsulating ammonium sulphate

**Fig. 1:**
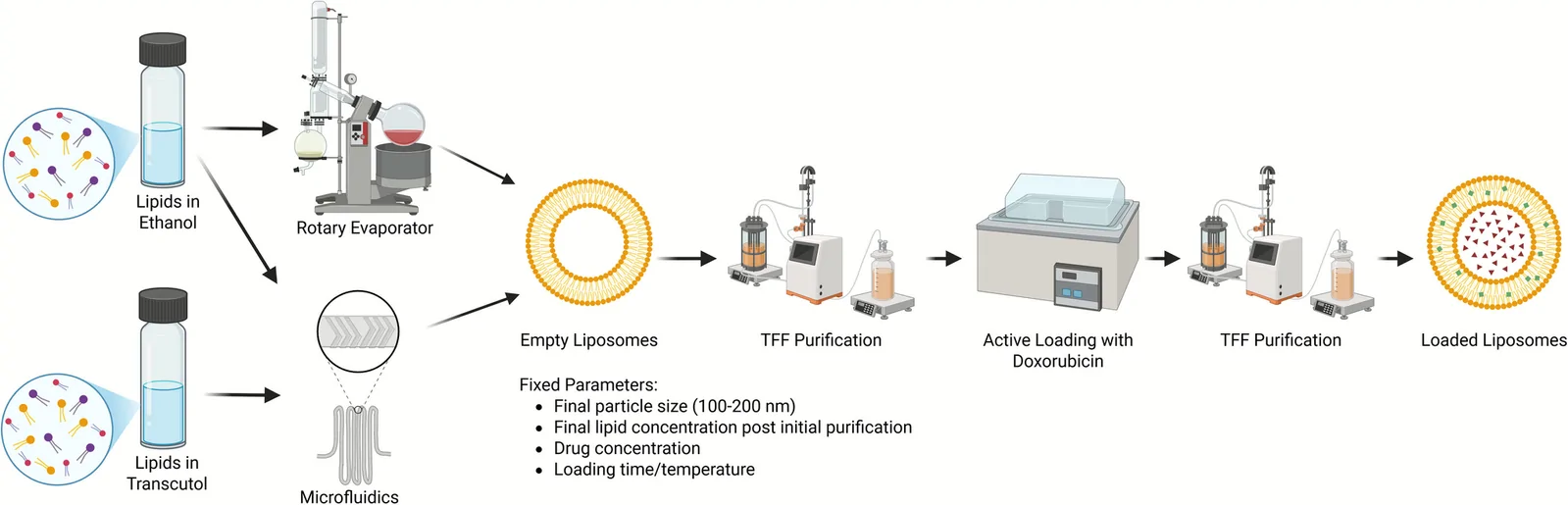
Liposomal Doxorubicin Manufacturing and Purification Process. DSPC:Cholesterol:DSPE-PEG2000 (w/w 3:1:1) liposomes were manufactured from either an ethanol lipid stock via Rotary Evaporator or microfluidics or from a Transcutol lipid stock via microfluidics only. The resultant liposomes were manufactured to be of particle size between 100–200 nm. All liposomes were purified to the same final lipid concentration (16 mg/mL) prior to remote loading with doxorubicin. All liposomes were loaded with the same concentration of doxorubicin at the same time and at the same temperature. After loading, all liposomes were again purified by TFF, and encapsulation was measured by UV absorbance. All samples were diluted to the same concentration for in vivo administration. Image created in BioRender https://BioRender.com/dospuk6

PEGylated liposomes were prepared using the solvent evaporation method (Fig. 1). DSPC, cholesterol (Chol), and DSPE-PEG2000 were dissolved in methanol at a total lipid concentration of 20 mg/mL (3:1:1 w/w), corresponding to an approximate molar ratio of 10.6:7.2:1 (DSPC:Chol:DSPE-PEG2000). The lipid solution was transferred to a round-bottom flask and the solvent removed under vacuum using a rotary evaporator. The resulting thin lipid film was rehydrated in 50 mL of 250 mM ammonium sulphate ([NH_4_]_2_SO_4_), heated to 60 °C, and vortexed until no visible lipid residue remained on the flask wall, as assessed by visual inspection. No additional analytical method was used to confirm the absence of residual lipid at this stage. The suspension was then processed by sequential extrusion (Liposo-Fast™−50, Avestin Inc., Ottawa, Canada) through polycarbonate filters of decreasing pore sizes (0.8, 0.4, and 0.2 µm), followed by sonication (3 cycles, 30 s on/30 s off), yielding liposomes with a final particle size of 90–100 nm.

### Buffer exchange and gradient formation for active loading

PEGylated liposomes encapsulating 250 mM ammonium sulphate [NH_4_]_2_SO_4_ were produced, and the organic solvent was removed while simultaneously performing buffer exchange to establish a transmembrane pH gradient for active doxorubicin loading. Tangential flow filtration (TFF) was performed using a KR2i TFF System (Repligen, Waltham, US) fitted with a 500 kDa modified polyethersulfone (mPES) column and Masterflex tubing 13. The suspension was processed at a flow rate of 20 mL/min for 12 diafiltration volumes against PBS (10 mM, pH 7.3). Before filtration, samples were diluted 1:5 with ammonium sulphate and then concentrated to a final lipid concentration of 20 mg/mL. This procedure yielded PEGylated liposomes containing an acidic core ([NH_4_]_2_SO_4_, pH 5.5) suspended in neutral PBS (pH 7.3), thereby generating a pH gradient suitable for active drug loading. Liposomes were subsequently diluted in PBS before sizing and active loading with doxorubicin.

### Active-loading of doxorubicin into PEGylated liposomes

Liposomes produced using microfluidics and rotary evaporation were purified using TFF as outlined. A doxorubicin stock solution (10 mg/mL in deionised water) was added to the liposome suspension at a drug-to-lipid ratio of 0.125 g:1 g (Fig. 1). The mixture was incubated in a water bath at 60 °C for 40 min to enable active loading, after which the formulations were cooled to room temperature and the non-encapsulated drug was removed by TFF using the same membrane cut-off, flow rate, and diafiltration conditions as described above. Encapsulation efficiency was quantified by UV absorbance at 490 nm using a microplate reader (Bio-Rad Laboratories Inc., Hertfordshire, UK). For analysis, liposome samples were mixed with 50% isopropyl alcohol to disrupt the bilayer and release the encapsulated drug. Calibration curves were prepared under identical conditions for each batch.

### USP-4 dissolution release study

Doxorubicin release was assessed using a USP-4 apparatus (SOTAX^®^, Allschwil, Switzerland) following the optimised method of Yuan et al. [14]. The assay was performed in a closed loop at a flow rate of 16 mL/min and at 45 °C. The release medium was freshly prepared and contained 100 mM ammonium bicarbonate (NH_4_HCO_3_), 75 mM 2-(N-morpholino)ethane sulfonic acid (MES), 5% w/v hydroxypropyl-β-cyclodextrin (HP-β-CD) to prevent doxorubicin precipitation, and 5% w/v sucrose. A dialysis membrane with a 300 kDa cut-off (Repligen, Waltham, US) was used. Each cell contained 39.2 mL of release medium and 0.8 mL of liposome suspension, diluted to a final doxorubicin concentration of 1 mg/mL. Free doxorubicin (1 mg/mL) was included as a control in each run. Aliquots were collected at 0.5, 1, 2, 3, 4, and 6 h. Cumulative release was calculated as described by Yuan et al. [14], expressed as the percentage of doxorubicin released from liposomes at each time point relative to the free drug control. Absorbance was measured at 490 nm using a UV plate reader (300 µL/well; Bio-Rad Laboratories Inc., Hertfordshire, UK). Release testing was performed using a USP-4 apparatus rather than biological media, as actively loaded liposomal doxorubicin formulations are well established to exhibit negligible drug release in serum-containing environments, with release governed primarily by carrier clearance rather than premature drug leakage. This behaviour has been previously demonstrated for actively loaded PEGylated liposomal doxorubicin, where drug release in biological media is minimal over relevant timescales [2, 14].

### In vivo biodistribution study

Male Sprague–Dawley rats were divided into 6 groups (3 groups of 9 rats, 3 rats sacrificed at each time point and 3 groups of 6 rats, all sacrificed at the same time point) and were administered doxorubicin-loaded liposomes via intravenous tail vein injection at a dose of 1 mg/kg. This dose was selected to minimise toxicity while maintaining quantifiable plasma and tissue concentrations, enabling sensitive detection of formulation- and manufacturing-related differences in biodistribution rather than dose-driven saturation effects. Liposomes were produced by either rotary evaporation or microfluidics using ethanol or Transcutol as the organic solvent. At four predetermined time points, rats were anesthetised, and blood and organs/tissues were collected. Blood was collected by heart puncture, and the plasma was separated by centrifugation for 10 min at 4 °C at 1900 g. All specimens were stored at −20 °C until analysed.

### LC–MS analysis of doxorubicin biodistribution in plasma and tissues

Plasma samples were mixed 1:1 with 0.1% formic acid in acetonitrile and centrifuged at 15,000 g for 30 min at 4 °C. The supernatant was transferred to a clean tube, filtered through a 0.22 µm PES filter, spiked with 800 ng of internal standard (daunorubicin), and analysed by LC–MS. Chromatographic separation was achieved on a Phenomenex Kinetex C18 column (50 × 3 mm, 2.6 µm) maintained at 30 °C. The mobile phase consisted of 0.1% formic acid (v/v) in water and acetonitrile, delivered at 0.4 mL/min using the following gradient: 5% acetonitrile from 0–8 min, increased to 40% from 8–9 min, then returned to 5% at 9.2 min. The injection volume was 1 µL. A Nexera-i LC-2040C system, coupled to an LCMS-8050 mass spectrometer (Shimadzu), equipped with an electrospray ionisation source operated in positive ion mode, was used. Ion pairs monitored were m/z 544.2 → 397.2 for doxorubicin and m/z 528.1 → 321.1 for daunorubicin. Frozen tissue samples (liver, spleen, kidney, lung, and heart) were weighed and homogenised in mobile phase (4 mL/g) using a LabGEN 7B homogeniser (Cole Palmer). Homogenates were mixed 1:1 with 0.1% formic acid in acetonitrile and centrifuged at 15,000 g for 30 min at 4 °C. Supernatants were filtered through a 0.22 µm filter, spiked with 800 ng daunorubicin, and analysed as described above.

The LC–MS method was adapted from previously published methods for the quantification of doxorubicin in plasma and tissue matrices [15, 16]. Quantification was performed using external calibration curves prepared in matched matrices over a linear concentration range of 5–8000 ng/mL, with daunorubicin used as an internal standard. Linearity was confirmed over this range (R^2^ > 0.99). As the method was adapted from established literature rather than newly developed, full bioanalytical validation was not repeated.

### Statistical analysis

For comparative exposure analysis, area under the concentration–time curve (AUC) values were calculated using the trapezoidal method over the measured time interval and used to support comparative assessment of systemic and tissue exposure rather than formal pharmacokinetic modelling. Statistical comparisons of AUC were carried out on AUC-derived metrics only. Minitab was used to perform a normality test (Anderson–Darling) to determine whether the data were normally distributed prior to further statistical analysis. A one-way ANOVA followed by Tukey’s multiple comparison test was used for data analysis. All experiments were carried out at least in triplicate, unless otherwise stated, and are presented as the mean of the measurements ± standard deviation, with error bars indicating the standard deviation. For the statistical analysis of the drug release studies from various formulations, the f2 similarity test was followed.

### Ethics approval

The animal study was approved by the animal ethics committee of the institution in accordance with the European Convention for the Protection of Vertebrate Animals Used for Experimental and Other Scientific Purposes (ETS no. 123) and followed the Belgian Royal Decree of May 29, 2013, for the Protection of Laboratory Animals.

## Results

### Physicochemical profiles of microfluidic liposomes (Ethanol vs Transcutol)

Microfluidic mixing offers a one-step process that generates liposomes with controlled size and low polydispersity, whereas rotary evaporation requires extrusion and sonication to reduce heterogeneity, increasing processing time and risking lipid degradation [17]. To assess whether solvent choice in microfluidic manufacture represents a critical parameter, liposomes were prepared using ethanol (Ethanol MF) or Transcutol (Transcutol MF) as solvents and compared with liposomes prepared by a conventional rotary evaporator protocol (Rotary Evap). Doxorubicin was selected as a benchmark compound given its extensive use in preclinical and clinical studies. As liposomal pharmacokinetics and pharmacodynamics are strongly influenced by vesicle size, drug loading, and release behaviour [18], liposomes were manufactured with comparable characteristics in terms of size and drug loading. Process parameters for microfluidic manufacture were selected based on our previous studies, which systematically examined the effect of formulation and microfluidic variables on liposome characteristics [13]. The rotary evaporation process was also optimised to achieve comparable properties (Figure S1).

**Fig. 2:**
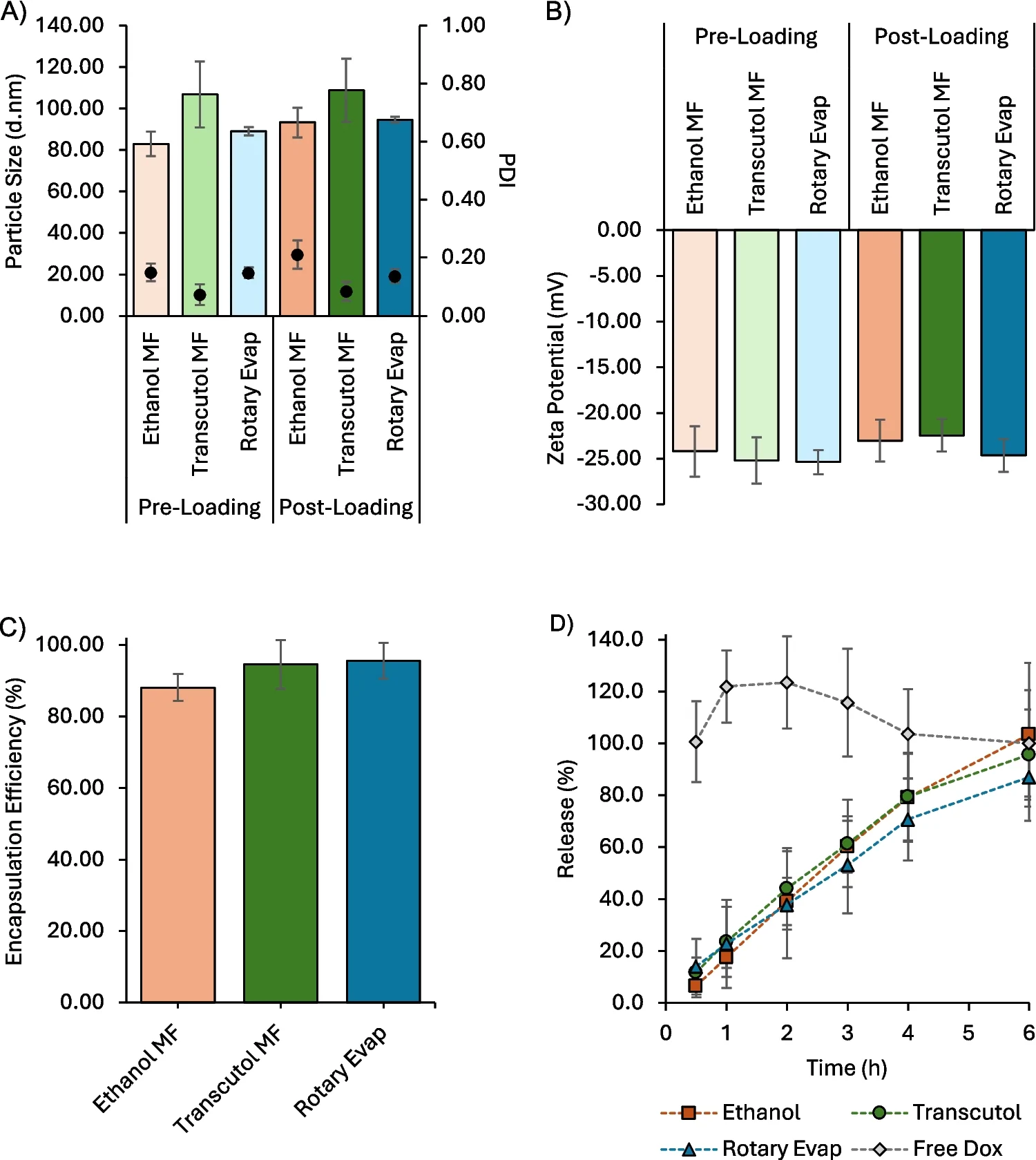
Physicochemical characterisation and in vitro release profiles of liposomal doxorubicin prepared using microfluidic mixing or conventional rotary evaporation. **A**) particle size (d.nm; bars) and PDI (discrete points) measured before and after doxorubicin loading. **B**) zeta potential of liposomes measured before and after doxorubicin loading. **C**) Encapsulation efficiency following drug loading. **D**) In vitro release profiles of free doxorubicin and liposomal formulations produced by microfluidics using ethanol (Ethanol MF) or Transcutol (Transcutol MF), or by rotary evaporation (Rotary Evap), determined using the USP-4 apparatus. Release studies were carried out at 45 °C in release media composed of 100 mM NH_4_HCO_3_, 75 mM 2-(N-morpholino)ethane sulfonic acid (MES), 5% w/v hydroxypropyl-β-cyclodextrin (HP-β-CD), and 5% w/v sucrose. Results represent mean ± SD, n = 5 independent batches

Using these optimised processes [13] (Figure S1), we were able to produce nanosized liposomes (approximately 80–110 nm) with PDI values < 0.2 and no statistically significant differences in size or homogeneity between groups before doxorubicin loading (Fig. 2A). After doxorubicin loading, the particle size increased by approximately 5–10 nm for the ethanol MF and rotary evaporator liposomes, whereas Transcutol MF formulations showed no measurable change (Fig. 2A). Zeta potential values were similar across all formulations both before and after drug loading (Fig. 2B), and encapsulation efficiency exceeded 85% for all formulations with no significant differences observed (Fig. 2C). Drug release studies (Fig. 2D) showed that free doxorubicin diffused completely within 0.5 h, whereas liposomal formulations released the drug gradually, with approximately 10 to 20% release at 0.5 h and complete release by 6 h (Fig. 2D). Release profiles did not differ significantly among Ethanol MF, Transcutol MF, and Rotary Evap formulations (F2 > 50), indicating that manufacturing method and solvent selection did not affect in vitro release behaviour. Taken together, the results in Fig. 2 show that, irrespective of the solvent used in microfluidics, liposomes were produced with comparable size, PDI, zeta potential and release behaviour for liposomal doxorubicin under USP4 conditions. To determine whether differences not evident in vitro emerged in vivo, we next assessed biodistribution following intravenous administration.

### In vivo biodistribution of liposomal doxorubicin: ethanol vs transcutol microfluidics in rats

**Fig. 3:**
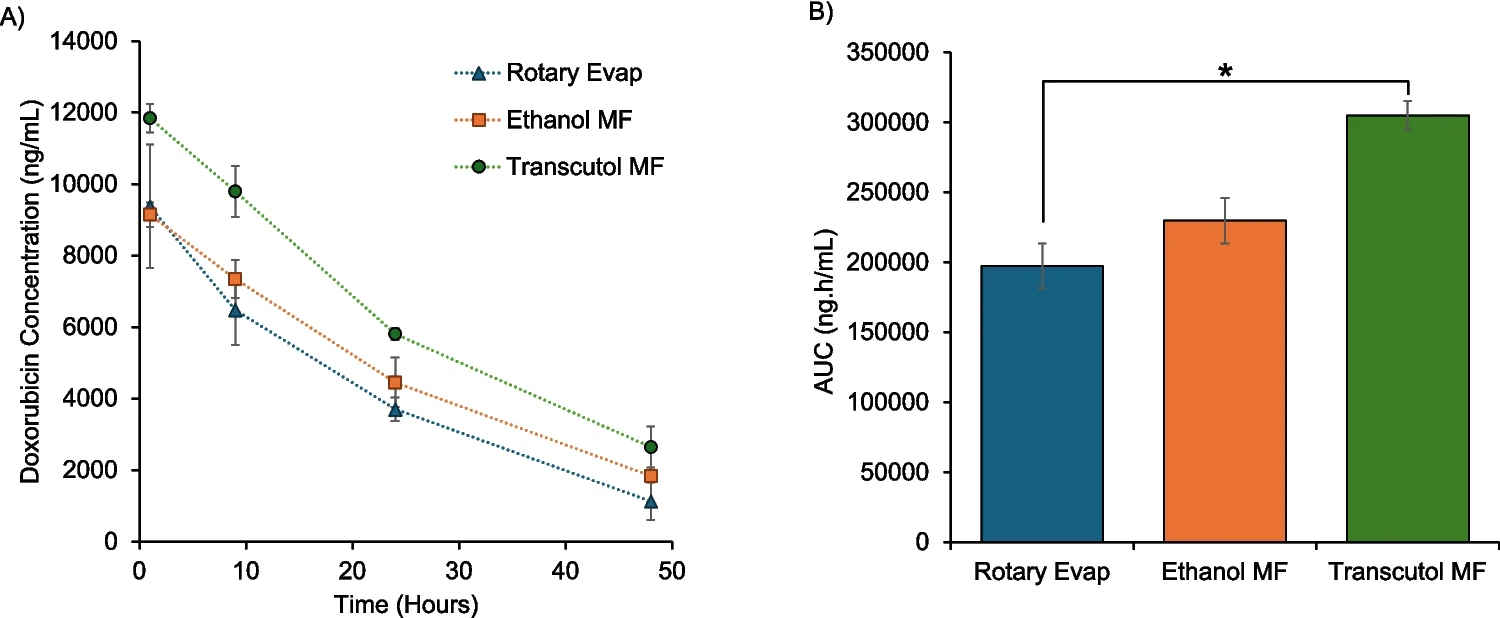
Plasma pharmacokinetics of liposomal doxorubicin in rats. **A**) Plasma concentration–time profiles following i.v. administration (1 mg/kg) of PEGylated liposomes prepared by microfluidics with ethanol (squares) or Transcutol (circles), or by rotary evaporation (triangles). Plasma samples were collected at 1, 9, 24, and 48 h. **B**) Corresponding plasma exposure (AUC₀–48 h) for each formulation. Data are presented as mean ± SD from independent animals at each time point (*n* = 3), except at 48 h, where values represent mean ± SD for n = 6 independent samples. Statistical comparisons were performed using one-way ANOVA with appropriate post hoc testing; *p* < 0.05 was considered statistically significant and indicated where appropriate

Having established comparable in vitro CQAs and release profiles (Fig. 2), we advanced all three formulations to in vivo evaluation. Male Sprague–Dawley rats received an intravenous dose of 1 mg/kg; plasma and tissues were sampled at 1, 9, 24, and 48 h post-administration. Plasma concentration–time profiles (Fig. 3) showed the expected monotonic decline for all formulations (Fig. 3A); however, liposomes produced using Transcutol (Transcutol MF) as the solvent had significantly higher concentrations than the rotary evaporator formulation at all time points, and significantly higher concentrations than the ethanol formulation at 1 h post-injection (*p* < 0.05). Consistent with these observations, area under the curve (AUC) analysis showed that liposomes prepared using Transcutol during microfluidics were not significantly different from liposomes prepared using ethanol (Ethanol MF), but exhibited a significantly higher AUC (*p* < 0.05) than liposomes prepared by rotary evaporation (Rotary Evap; Fig. 3B).

**Fig. 4:**
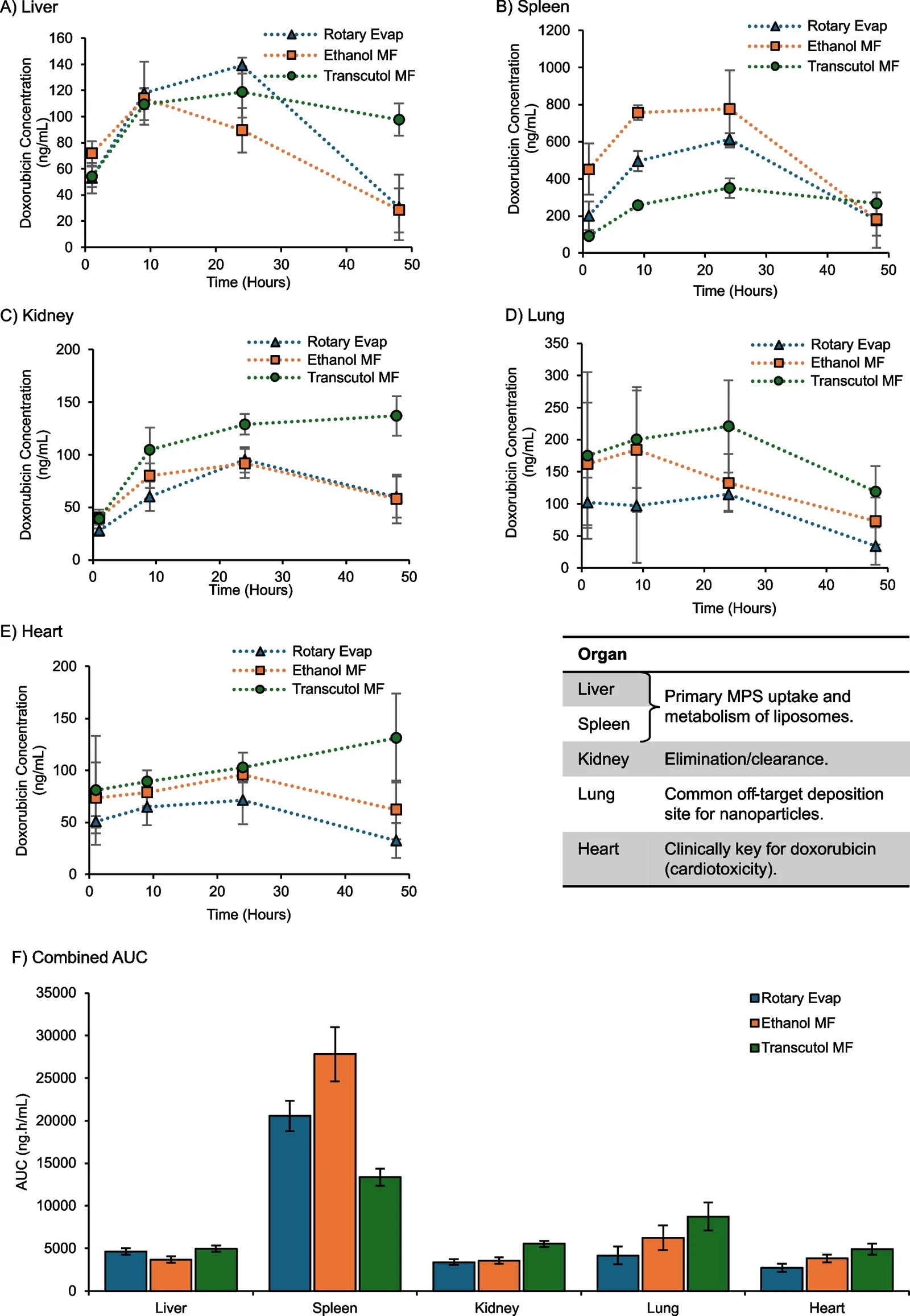
Tissue concentration vs time profiles of doxorubicin following intravenous administration of liposomal formulations in rats. Liposomes were produced by rotary evaporation or by microfluidic mixing using ethanol or Transcutol as the organic solvent. Time–concentration profiles of doxorubicin in **A**) liver, **B**) spleen, **C**) kidney, **D**) lung and **E**) heart following intravenous administration (1 mg/kg) of PEGylated liposomes prepared by microfluidics with ethanol (squares) or Transcutol (circles), or by rotary evaporation (triangles). **F**) Area under the concentration–time curve (AUC₀–48 h) for doxorubicin measured in liver, spleen, kidney, lung, and heart. Male Sprague–Dawley rats were sampled at 1, 9, 24 and 48 h post-administration. Results represent mean ± SD, from independent animals (*n* = 3 per time point), except at 48 h, where results represent mean ± SD from n = 6 of independent samples. The sidebar outlines the physiological context of each organ (MPS uptake/metabolism in liver and spleen; renal elimination; lung deposition; clinically relevant heart exposure)

Building on the plasma PK, we asked whether the choice of solvent during microfluidic manufacturing results in distinct tissue distribution patterns. Figure 4 shows doxorubicin time-concentration profiles in the liver, spleen, kidney, lung and heart after intravenous dosing (1 mg/kg). Across organs, all three formulations exhibited broadly similar overall exposure over the 48 h sampling window for most tissues; however, formulation-dependent differences were evident at specific time points, indicating temporal redistribution of drug rather than major differences in overall tissue uptake or clearance.

In the liver (Fig. 4A), profiles were comparable for the first 24 h, and by 48 h, rats receiving the Transcutol MF formulations maintained higher doxorubicin concentrations than rats receiving the Rotary Evap and Ethanol MF formulations. In the spleen (Fig. 4B), the Transcutol MF formulation tended to yield lower doxorubicin levels, whereas kidney (Fig. 4C) and lung (Fig. 4D) drug concentrations were generally higher relative to the other formulations; heart exposures remained low and similar across formulations (Fig. 4E). The AUC profiles summaries (Fig. 4F) reinforce these trends. AUCs were not significantly different between formulations in the liver, kidney, lung or heart, while the spleen showed a significantly lower AUC (*p* < 0.05) in rats treated with Transcutol MF than in those treated with the Ethanol MF or Rotary Evap formulation. Notably, these in vivo differences arise despite similar physicochemical characteristics and in vitro release profiles (Fig. 2), highlighting solvent selection during manufacture as a determinant of distribution beyond bulk CQAs. In contrast, when comparing the rotary evaporator formulation with those produced via microfluidics using ethanol, no significant differences were observed, indicating close equivalence between these two manufacturing approaches.

**Fig. 5:**
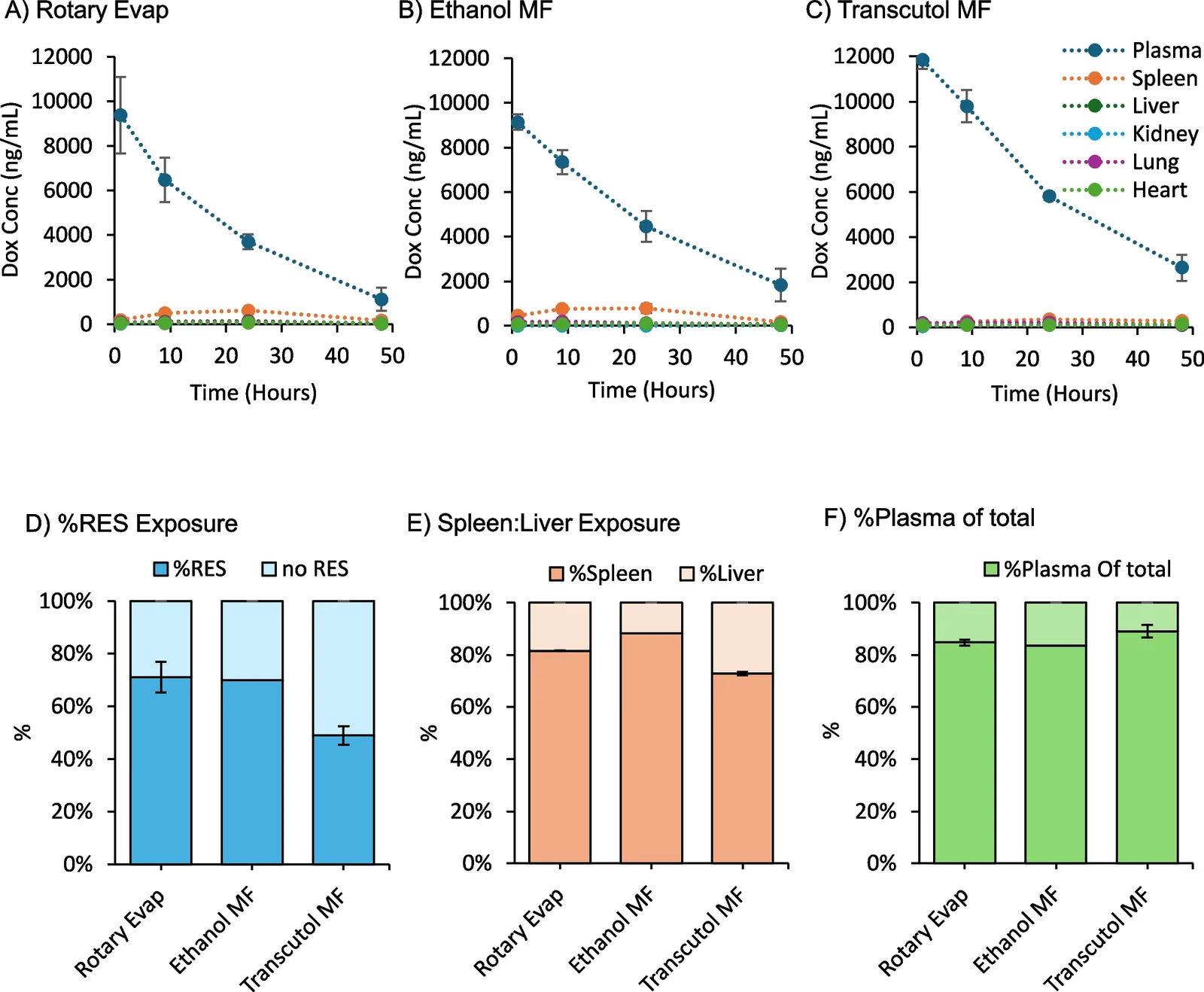
Formulation-dependent biodistribution of liposomal doxorubicin in rats. Combined concentration–time profiles across tissues following intravenous administration of liposomes produced by **A**) rotary evaporation, **B**) microfluidic mixing using ethanol, and **C**) microfluidic mixing using Transcutol. **D**) Percentage exposure of each formulation to the primary reticuloendothelial system (RES) organs (liver + spleen, expressed as a fraction of total tissue AUC). **E**) Percentage allocation within the RES, expressed as the spleen:liver exposure ratio. **F**) Percentage partition of doxorubicin between the plasma and tissues. Results represent mean ± SD from independent animals (*n* = 3 per time point), except at 48 h, where results represent mean ± SD from n = 6 independent samples

To complement the organ-centric view in Fig. 4, we re-plotted the same biodistribution data grouped by formulation (Fig. 5). This per-formulation view enables direct, within-formulation comparisons across organs, highlighting that the highest doxorubicin concentrations were observed in plasma for all formulations, whereas peripheral organs had comparatively low levels. When taken together, the rotary evaporator and the ethanol formulations show very similar biodistribution profiles (Fig. 5A and B), demonstrating that it was possible to translate liposome production from more traditional manufacturing methods (i.e., rotary evaporator) to more scalable methods such as microfluidics without altering in vivo behaviour. The results also illustrate how the manufacturing parameters can influence liposome performance. Although both the ethanol and Transcutol formulations were produced using microfluidics and manufactured to have similar particle characteristics after doxorubicin loading (Fig. 2), subtle differences in biodistribution profiles were observed (Figs. 4 and 5). Liposomes produced from Transcutol maintained higher plasma concentrations for longer than the ethanol formulation and showed reduced accumulation in the spleen and reduced clearance in the liver, kidney, lung, and heart (Figs. 4 and 5).

To further consider the organ time courses, we derived percentage RES exposure (liver + spleen share of total tissue AUC; Fig. 5D), spleen:liver exposure ratio (Fig. 5E), and percentage plasma of total exposure (plasma AUC as a fraction of the summed plasma + tissue AUCs; Fig. 5F). Rotary Evap and Ethanol MF were essentially indistinguishable across all three metrics, whereas Transcutol MF diverged in some specific parameters (Fig. 5D–F). Specifically, percentage RES exposure was similar for Rotary Evap and Ethanol MF (~ 71% and ~ 70%, respectively) but substantially lower for Transcutol MF (approximately 49%), indicating reduced allocation to primary RES organs (Fig. 5D). The spleen:liver ratio was also lower for Transcutol MF, reflecting reduced relative splenic exposure (Fig. 5E). In contrast, the percentage plasma exposure was similar for all three formulations (Fig. 5F). These results again suggest that Transcutol-based microfluidic assembly of the liposomal formulations shifts exposure away from the RES and towards circulating plasma. At the same time, ethanol-based microfluidics closely matches the rotary-evaporated comparator.

## Discussion

In this paper, we asked two linked questions: first, can microfluidic manufacturing generate liposomes with characteristics comparable to those prepared by conventional manufacturing protocols, and second, within microfluidics, does solvent choice (ethanol vs. Transcutol) serve as a critical material attribute that alters performance? This framing reflects the fact that relatively minor process differences can have a major impact on liposomal attributes [14]. Liposomes are complex delivery systems with heterogeneous structures and varying physicochemical properties, and establishing the extent of similarity is therefore essential when comparing new formulations with reference products [19]. Previous research has shown that liposome critical quality attributes can be tailored by varying microfluidic manufacturing parameters (i.e. flow rates and mixing ratios [2]), and that liposome formulation design choices, such as PEGylation, can alter biodistribution (e.g. Doxil/Caelyx vs Myocet) [20]. In this context, our results support a simple yet important conclusion: when bulk CQAs and in vitro release are comparable, solvent choice and process conditions during microfluidic assembly can still imprint distinct in vivo distribution patterns. This highlights a potential gap in current “CQA-matching” paradigms, where subtle structural features not captured by routine assays may nevertheless drive biological behaviour.

Several factors can affect drug release from liposomal formulations, including membrane composition, size distribution, lamellarity, the nature and physical state of the drug within the liposome, and the dominant release mechanism [21, 22]. Membrane composition is a key determinant of release kinetics: phospholipid Tm (set by hydrocarbon chain length and saturation) and helper lipid/cholesterol content modulate permeability [23, 24]. Because lipid composition was held constant in the present study (DSPC/Chol/DSPE-PEG2000), the observed differences are unlikely to arise from lipid identity per se. Even with matched composition, however, small changes in manufacturing conditions can influence release behaviour. For example, USP-4 methods have been shown to discriminate between distinct liposomal formulations and generic doxorubicin products, and to differentiate POPC from HSPC systems based on fluidity at 45 °C versus > 55 °C [14]. These studies also support USP-4 as a comparative tool for assessing doxorubicin release from generics against Doxil^®^ [14].

The lack of separation in our USP-4 data (Fig. 2) therefore points away from bulk permeability differences and toward higher-order structural effects, such as altered acyl-chain packing, interleaflet coupling, or lamellarity. This interpretation is consistent with reports of slower release from multilamellar systems, where additional bilayers hinder diffusion [25], and with observations that solvent choice during microfluidics can bias vesicle architecture (e.g. ethanol promotes SUVs; whereas isopropanol yields double-bilayer vesicles) [26]. During assembly, solvent–lipid interactions can reconfigure chain ordering and bilayer packing; for example, alkanols such as ethanol can intercalate near headgroups and modulate Tm in a chain-length–dependent manner [27]. Although residual solvent after TFF is typically low (< 0.3%) [28], even brief exposure during formation may still influence bilayer organisation [29]. In our experiments, Transcutol liposomes were prepared at a lower total flow rate and consequently longer effective contact time than for ethanol to ensure matched particle sizes (5 mL/min for Transcutol versus 10 mL/min for ethanol), potentially influencing bilayer packaging. While not evident in vitro by USP-4, such architectural differences could affect opsonisation and organ allocation in vivo. However, direct ultrastructural characterisation of the liposomes (e.g. by electron microscopy or scattering-based techniques) was not performed as part of this study and is acknowledged as an important direction for future work. Accordingly, the present findings highlight manufacturing-dependent effects on in vivo behaviour without assigning a definitive structural mechanism.

During microfluidic production, solvent selection has been shown to affect liposome formation [26], with the rate of change in polarity influencing particle size; size, in turn, can shape biodistribution (100–150 nm favouring uptake; 50–100 nm is reported to be better at avoiding the immune system) [30]. Here, production was optimised so that all PEGylated formulations were similar in size (≈100–120 nm; Fig. 2), and PEGylated systems within the 80–250 nm range generally show reduced size sensitivity relative to non-PEGylated liposomes [31]. The modest increase in liposome size post-drug-loading (Fig. 2) is consistent with doxorubicin complexation with sulfate in the core and the formation of rod-like structures spanning the vesicle diameter, which can slightly shift morphology and PDI [32]. While size can modulate release independently of composition (e.g., ~ 40% release at 240 min for 50 nm vs none for 100 nm daunorubicin liposomes [33], the absence of both significant sizes differences and USP-4 separation in the present study suggests that size is unlikely to be the primary driver.

The in vivo pattern (higher plasma exposure with reduced early liver/spleen accumulation for Transcutol-assembled liposomes; Figs. 3, 4, 5) aligns with solvent-conditioned membrane organisation rather than with bulk permeability differences. Formation-stage solvent exposure can imprint bilayer architecture [27, 29], and even brief solvent exposure during the formation stage may still template bilayer architecture (e.g., conversion from unilamellar to multilamellar structures). In our study, the Transcutol manufacturing process, which gave longer solvent contact, could plausibly favour alternative packing/lamellarity that redirects RES engagement. Consistent with this, plasma enrichment may reflect greater vesicle stability, slower release, and/or diminished cellular uptake with Transcutol-assembled liposomes [36]. Taken together, these observations are consistent with solvent-conditioned effects arising during assembly that influence in vivo behaviour, despite matched lipid composition and similar in vitro release.

From a translational and regulatory perspective, these findings have important implications for assessing nanomedicine comparability, particularly in the context of off-patent “nanosimilar” development. Nanomedicines are often recognised as non-biological complex drugs, for which quality, safety and efficacy are intrinsically linked to higher-order structure and the manufacturing process itself [34, 35]. Therefore, regulatory frameworks emphasise a weight-of-evidence approach to comparability, integrating physicochemical characterisation with relevant biological and pharmacokinetic data rather than relying solely on conventional CQAs. Within a quality-by-design framework, the present results further suggest that solvent selection during microfluidic manufacturing should be considered a formulation-relevant variable. While solvent choice is often adjusted to address lipid solubility and can be used to achieve target particle size profiles (e.g. [13]), solvent-lipid interactions and effective solvent contact time during assembly may influence higher-order attributes that are not routinely captured by standard assays. From a design-space perspective, solvent identity and associated process parameters (e.g., solvent fraction, total flow rate, and mixing conditions) may function as critical material attributes or critical process parameters, whose acceptable ranges should be justified not only on the basis of physicochemical outputs but also on their potential impact on biological performance. In this context, solvent identity may be regarded as a material attribute, while solvent fraction and solvent-lipid interaction time during microfluidic assembly may act as critical process parameters, with their classification depending on how solvent selection is implemented within the design space. Explicit incorporation of solvent choice into QbD strategies may therefore improve the robustness of comparability assessments, particularly when transitioning between manufacturing routes or during the development of nanosimiliar liposome products.

Importantly, the conclusions drawn here are based on convergent biological and formulation evidence, rather than direct structural measurements, which remain an important area for future investigation.

## Conclusion

These studies demonstrate that microfluidic manufacturing can deliver CQA-matched, doxorubicin-loaded PEGylated liposomes comparable to those from a conventional rotary evaporation/extrusion process, with similar size, polydispersity, zeta potential, encapsulation efficiency, and indistinguishable USP-4 release profiles. However, solvent choice within the microfluidic assembly emerged as a critical factor influencing in vivo disposition: formulations assembled with Transcutol showed greater plasma exposure and reduced early splenic accumulation, with organ-specific differences reflecting time-dependent variations in tissue distribution of the drug rather than fundamental changes in clearance kinetics, whereas ethanol-assembled liposomes closely tracked those prepared by rotary-evaporation. Because bulk CQAs and in vitro release were aligned, these in vivo shifts most plausibly reflect solvent-conditioned ultrastructure arising during formation (e.g., differences in bilayer packing/lamellarity) rather than composition or size alone. Together, these data demonstrate microfluidics as a route to clinically relevant liposomes, while identifying solvent selection as a parameter that must be explicitly incorporated into the design space for lipid-based nanomedicines. In practice, achieving CQAs is necessary but not sufficient, and manufacturing processes can affect biological performance. The partial divergence observed between in vitro release behaviour and in vivo pharmacokinetics does not negate the value of in vitro release testing in nanomedicine development; rather, it reinforces that in vitro release assays are a necessary, but not standalone, component of the development pipeline when manufacturing parameters differ.

## Supplementary Information

Below is the link to the electronic supplementary material.

Supplementary file1 (PDF 9771 KB)

## Data Availability

The data underpinning this publication is openly available from the University of Strathclyde KnowledgeBase at https://doi.org/10.15129/1ab826f7-196a-445f-abc6-2f1f113ffc6b.

## Declarations

### Data availability

The data underpinning this publication is openly available from the University of Strathclyde KnowledgeBase at https://doi.org/10.15129/1ab826f7-196a-445f-abc6-2f1f113ffc6b.
