# Supplementary material for: Impact of solvent choice during microfluidic manufacture on the in vivo performance of liposomal doxorubicin
Source: Drug Deliv Transl Res. 2026 Mar 2;16(10):3702–13. doi: 10.1007/s13346-026-02062-4 (PMC13619651; doi:10.1007/s13346-026-02062-4)
Supplement: Supplementary file 1 [file 13346_2026_2062_MOESM1_ESM.pdf]

SUPPLEMENTARY FIGURES

Title: Impact of Solvent Choice During Microfluidic Manufacture on the In Vivo Performance of Liposomal Doxorubicin.

Authors: Sarah Lindsay[1], René Holm[2], Sanket Shah[3] and Yvonne Perrie[1]

[1]Strathclyde Institute of Pharmacy and Biomedical Sciences, 161 Cathedral St, University of Strathclyde, Glasgow, G4 0RE, UK.

[2]Department of Physics, Chemistry, and Pharmacy, University of Southern Denmark, Campusvej 55, 5230 Odense, Denmark

[3]Therapeutics Development and Supply, Janssen Pharmaceutica NV, a Johnson & Johnson Company, Turnhoutseweg 30, 2340 Beerse, Belgium

Corresponding author:

Professor Yvonne Perrie

Strathclyde Institute of Pharmacy and Biomedical Sciences,

161 Cathedral St,

University of Strathclyde,

Glasgow, G4 0RE

UK.

yvonne.perrie@strath.ac.uk

A)

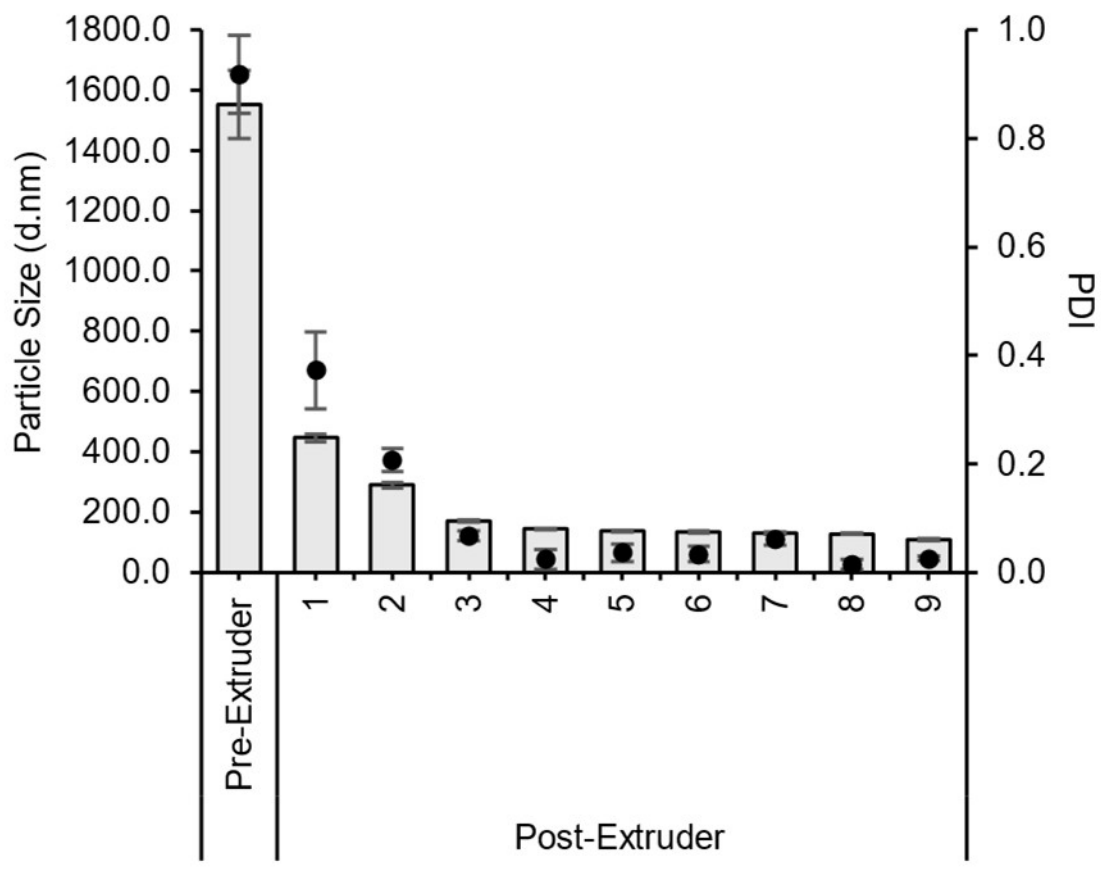

Post-Extrusion	Number of Passes	Number of filters	Pore Size (um)
1	2	2	1
2	2	2	0.4
3	2	2	0.2
4	2	1	0.1
5	2	2	0.1
6	2	3	0.1
7	4	2	0.1
8	8	3	0.1
9	2	1	0.05

B)

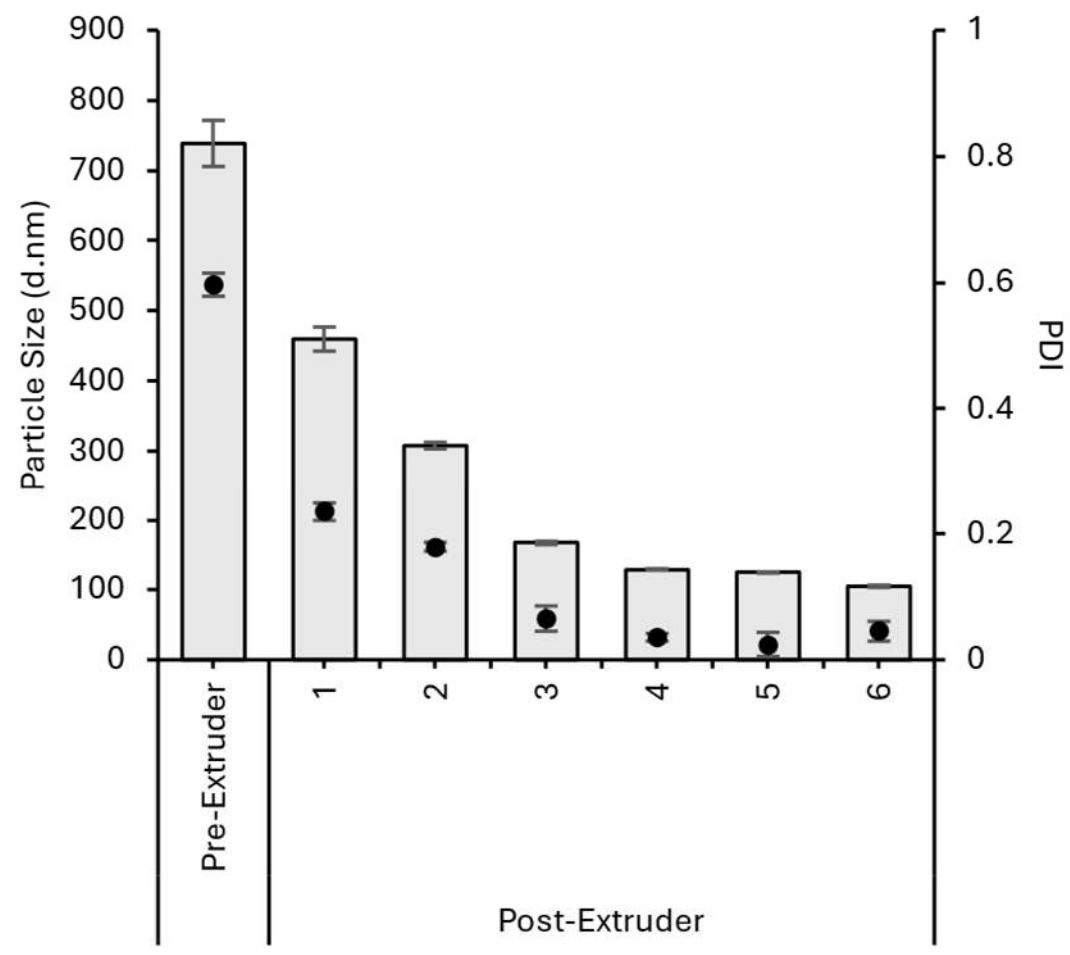

Post-Extrusion	Number of Passes	Number of filters	Pore Size (um)
1	1	2	1
2	1	2	0.4
3	2	2	0.2
4	5	2	0.1
5	1	3	0.1
6	3	1	0.05

C)

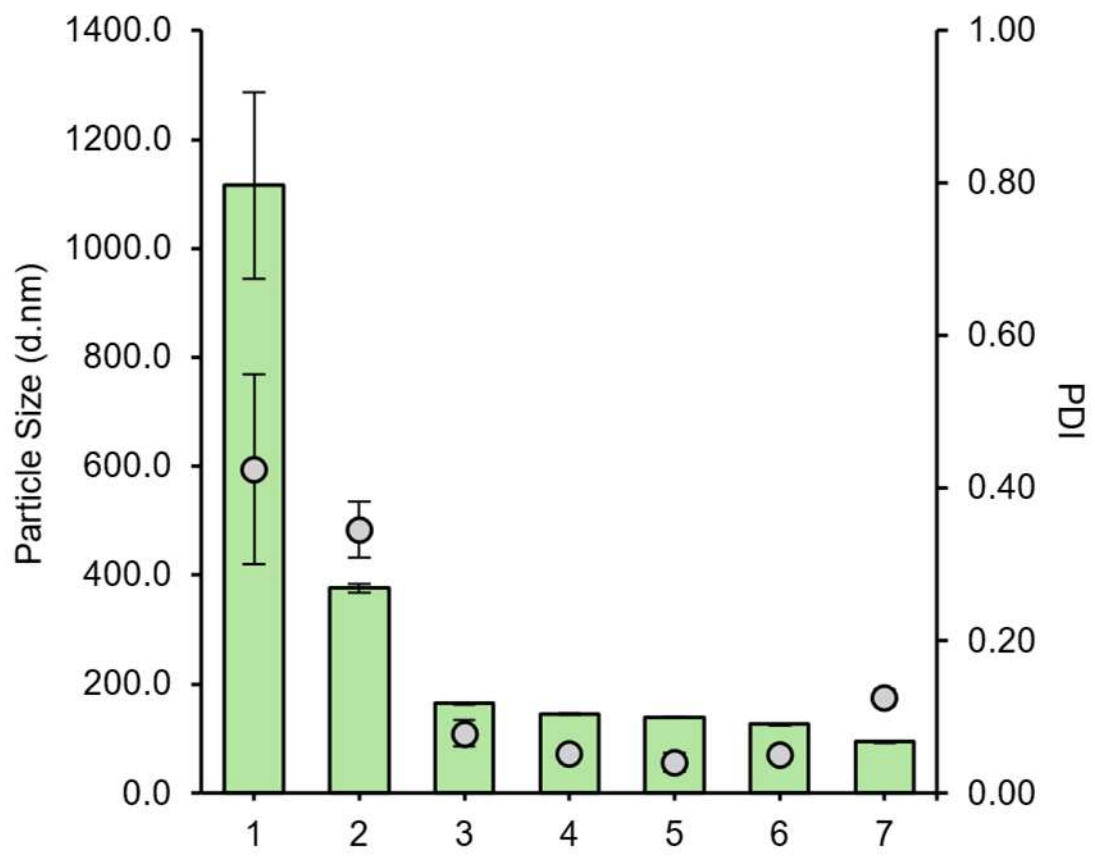

Sample	Number of Passes	Number of Filters	Pore Size (um)
1 (Pre-Extruder)	-	-	-
2	4	1	0.8
3	6	1	0.2
4	10	1	0.1
5	10	1	0.1
6	4	1	0.05
7 (Sonication)	3	30s on, 30s off	

Figure S1. Optimisation of liposome production method via rotary evaporation/lipid hydration and sonication. Liposomes were prepared by rotary evaporation, with the lipid film hydrated in 250 mM ammonium sulfate and then extruded through membranes of progressively decreasing pore size. Graphs show particle size (bars) and polydispersity index (PDI; discrete points) for liposomes after passage through the indicated membranes and after the indicated number of extrusion cycles, as detailed in the corresponding tables. A) Initial assessment of extrusion alone to determine the number of passes required to reduce particle size to approximately 100 nm. B) Further optimisation to evaluate whether the number of membranes and extrusion passes could be reduced while maintaining the target particle size. C) Particle size and PDI following extrusion with subsequent sonication to achieve additional size reduction; this condition was selected as the optimised protocol. Data are presented as mean ± SD (n = 3 repeated measurements of the same batch).
